# Control Centrality and Hierarchical Structure in Complex Networks

**DOI:** 10.1371/journal.pone.0044459

**Published:** 2012-09-27

**Authors:** Yang-Yu Liu, Jean-Jacques Slotine, Albert-László Barabási

**Affiliations:** 1 Center for Complex Network Research and Department of Physics,Northeastern University, Boston, Massachusetts, United States of America; 2 Center for Cancer Systems Biology, Dana-Farber Cancer Institute, Boston, Massachusetts, United States of America; 3 Nonlinear Systems Laboratory, Massachusetts Institute of Technology, Cambridge, Massachusetts, United States of America; 4 Department of Mechanical Engineering and Department of Brain and Cognitive Sciences, Massachusetts Institute of Technology, Cambridge, Massachusetts, United States of America; 5 Department of Medicine, Brigham and Women’s Hospital, Harvard Medical School, Boston, Massachusetts, United States of America; University of Zaragoza, Spain

## Abstract

We introduce the concept of control centrality to quantify the ability of a single node to control a directed weighted network. We calculate the distribution of control centrality for several real networks and find that it is mainly determined by the network’s degree distribution. We show that in a directed network without loops the control centrality of a node is uniquely determined by its layer index or topological position in the underlying hierarchical structure of the network. Inspired by the deep relation between control centrality and hierarchical structure in a general directed network, we design an efficient attack strategy against the controllability of malicious networks.

## Introduction

Complex networks have been at the forefront of statistical mechanics for more than a decade [1]–[4]. Studies of them impact our understanding and control of a wide range of systems, from Internet and the power-grid to cellular and ecological networks. Despite the diversity of complex networks, several basic universal principles have been uncovered that govern their topology and evolution [3], [4]. While these principles have significantly enriched our understanding of many networks that affect our lives, our ultimate goal is to develop the capability to control them [5]–[17].

According to control theory, a dynamical system is controllable if, with a suitable choice of inputs, it can be driven from any initial state to any desired final state in finite time [18]–[20]. By combining tools from control theory and network science, we proposed an efficient methodology to identify the minimum sets of driver nodes, whose time-dependent control can guide the whole network to any desired final state [12]. Yet, this minimum driver set (MDS) is usually not unique, but one can often achieve multiple potential control configurations with the same number of driver nodes. Given that some nodes may appear in some MDSs but not in other, a crucial question remains unanswered: what is the role of each individual node in controlling a complex system? Therefore the question that we address in this paper pertains to the importance of a given node in maintaining a system’s controllability.

Historically, various types of centrality measures of a node in a network have been introduced to determine the relative importance of the node within the network in appropriate circumstances. For example, the degree centrality, closeness centrality [21], betweenness centrality [22], eigenvector centrality [23], [24], PageRank [25], hub centrality and authority centrality [26], routing centrality [27], and so on. Here, we introduce control centrality to quantify the ability of a single node in controlling the whole network. Mathematically, control centrality of node 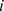 captures the dimension of the controllable subspace or the size of the controllable subsystem when we control node 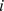 *only*. This agrees well with our intuitive notion about the “power” of a node in controlling the whole network. We notice that control centrality is fundamentally different from the concept of control range, which quantifies the “duty” or “responsibility” of a node 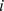 in controlling a network *together with other driver nodes* [28].

## Results

### Control Centrality

Consider a complex system described by a directed weighted network of 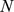 nodes whose time evolution follows the linear time-invariant dynamics.
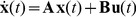
(1)where 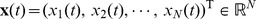 captures the state of each node at time 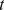. 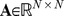 is an 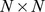 matrix describing the weighted wiring diagram of the network. The matrix element 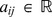 gives the strength or weight that node 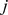 can affect node 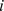. Positive (or negative) value of 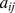 means the link 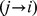 is excitatory (or inhibitory). 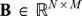 is an 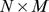 input matrix (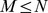) identifying the nodes that are controlled by the time dependent input vector 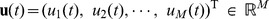 with 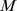 independent signals imposed by an outside controller. The matrix element 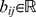 represents the coupling strength between the input signal 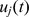 and node 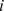. The system (1), also denoted as 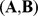, is controllable if and only if its controllability matrix 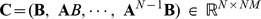 has full rank, a criteria often called Kalman’s controllability rank condition [18]. The rank of the controllability matrix 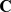, denoted by 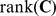, provides the dimension of the controllable subspace of the system 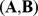 [18], [19]. When we control node 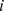 only, 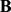 reduces to the vector 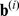 with a single non-zero entry, and we denote 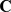 with 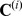. We can therefore use 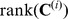 as a natural measure of node 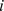
’s ability to control the system: if 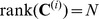, then node 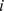 alone can control the whole system, i.e. it can drive the system between any points in the 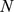-dimensional state space in finite time. Any value of 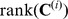 less than 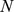 provides the dimension of the subspace 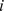 can control. In particular if 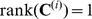, then node 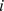 can only control itself.

The precise value of 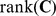 is difficult to determine because in reality the system parameters, i.e. the elements of 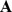 and 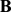, are often not known precisely except the zeros that mark the absence of connections between components of the system [29]. Hence 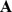 and 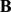 are often considered to be structured matrices, i.e. their elements are either fixed zeros or independent free parameters [29]. Apparently, 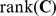 varies as a function of the free parameters of 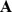 and 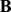. However, it achieves the maximal value for all but an exceptional set of values of the free parameters which forms a proper variety with Lebesgue measure zero in the parameter space [30], [31]. This maximal value is called the *generic rank* of the controllability matrix 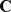, denoted as 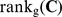, which also represents the generic dimension of the controllable subspace. When 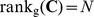, the system 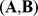 is *structurally controllable*, i.e. controllable for almost all sets of values of the free parameters of 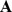 and 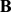 except an exceptional set of values with zero measure [29], [30], [32], [33]. For a single node 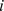, 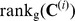 captures the “power” of 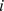 in controlling the whole network, allowing us to define the *control centrality* of node 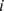 as
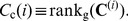
(2)


**Figure 1:**
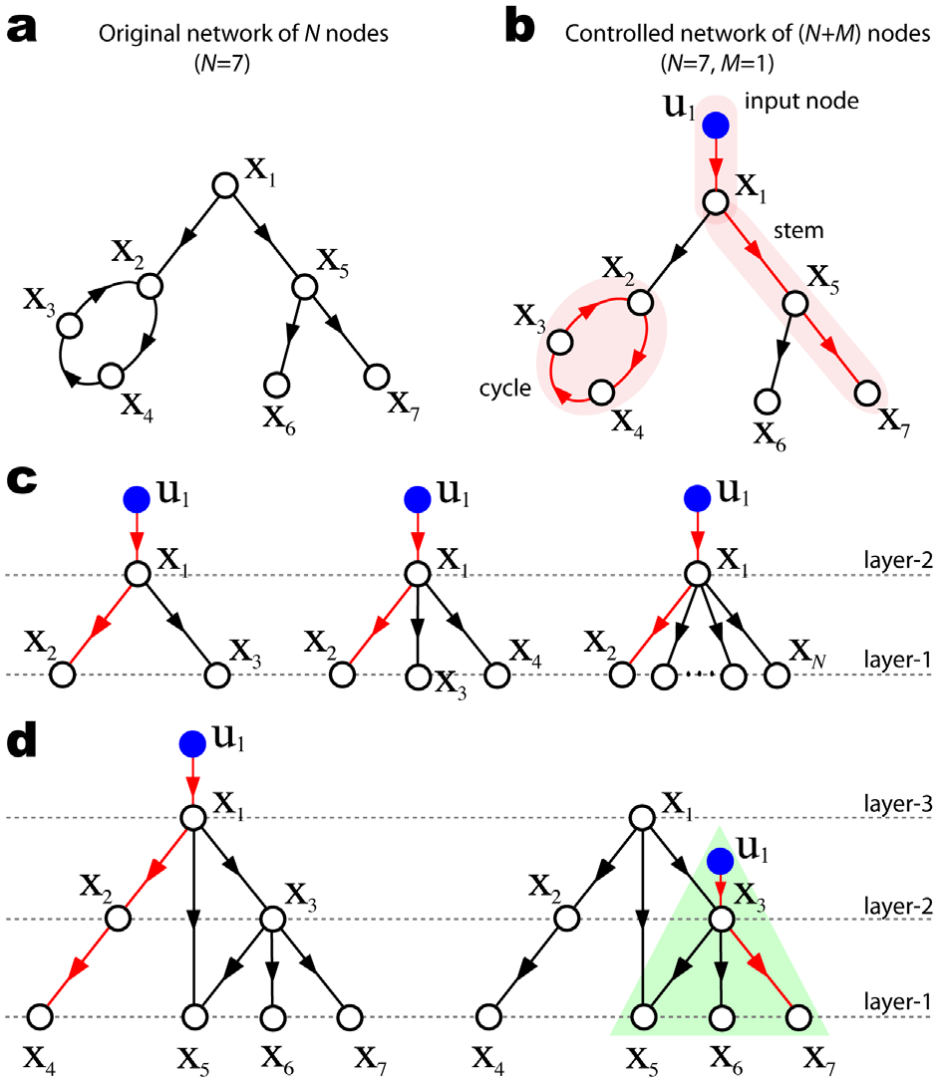
Control centrality. (a) A simple network of 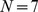 nodes. (b) The controlled network is represented by a directed graph 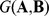 with an input node 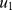 connecting to a state node 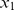. The stem-cycle disjoint subgraph 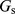 (shown in red) contains six edges, which is the largest number of edges among all possible stem-cycle disjoint subgraphs of the directed graph 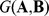 and corresponds to the generic dimension of controllable subspace by controlling node 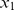. The control centrality of node 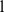 is thus 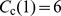. (c) The control centrality of the central hub in a directed star is always 2 for any network size 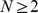. (d) The control centrality of a node in a directed acyclic graph (DAG) equals its layer index. In applying Hosoe’s theorem, if not all state nodes are accessible, we just need to consider the accessible part (highlighted in green) of the input node(s).



The calculation of 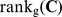 can be mapped into a combinatorial optimization problem on a directed graph 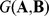 constructed as follows [31]. Connect the 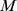 input nodes 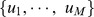 to the 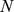 state nodes 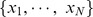 in the original network according to the input matrix 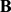, i.e. connect 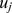 to 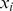 if 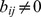, obtaining a directed graph 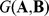 with 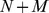 nodes (see Fig. 1a and b). A state node 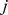 is called *accessible* if there is at least one directed path reaching from one of the input nodes to node 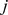. In Fig. 1b, all state nodes 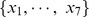 are accessible from the input node 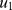. A *stem* is a directed path starting from an input node, so that no nodes appear more than once in it, e.g. 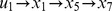 in Fig. 1b. Denote with 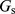 the *stem-cycle disjoint* subgraph of 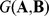, such that 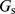 consists of stems and cycles only, and the stems and cycles have no node in common (highlighted in Fig. 1b). According to Hosoe’s theorem [31], the generic dimension of the controllable subspace is given by

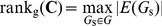
(3)with 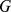 the set of all stem-cycle disjoint subgraphs of the accessible part of 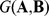 and 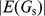 the number of edges in the subgraph 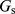. For example, the subgraph highlighted in Fig. 1b, denoted as 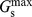, contains the largest number of edges among all possible stem-cycle disjoint subgraphs. Thus, 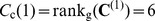, which is the number of red links in Fig. 1b. Note that 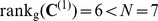, the whole system is therefore not structurally controllable by controlling 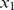 only. Yet, the nodes covered by the 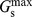 highlighted in Fig. 1b, e.g. 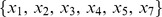, constitute a structurally controllable subsystem [33]. In other words, by controlling node 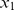 with a time dependent signal 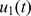 we can drive the subsystem 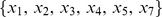 from any initial state to any final state in finite time, for almost all sets of values of the free parameters of 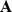 and 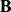 except an exceptional set of values with zero measure. In general 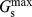 is not unique. For example, in Fig. 1b we can get the same cycle 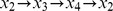 together with a different stem 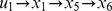, which yield a different 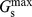 and thus a different structurally controllable subsystem 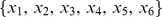. Both subsystems are of size six, which is exactly the generic dimension of the controllable subspace. Note that we can fully control each subsystem individually, yet we cannot fully control the whole system.

The advantage of Eq.(3) is that 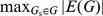 can be calculated via linear programming [34], providing us an efficient numerical tool to determine the control centrality and the structurally controllable subsystem of any node in an arbitrary complex network (see Fig. S1).

### Distribution of Control Centrality

**Figure 2:**
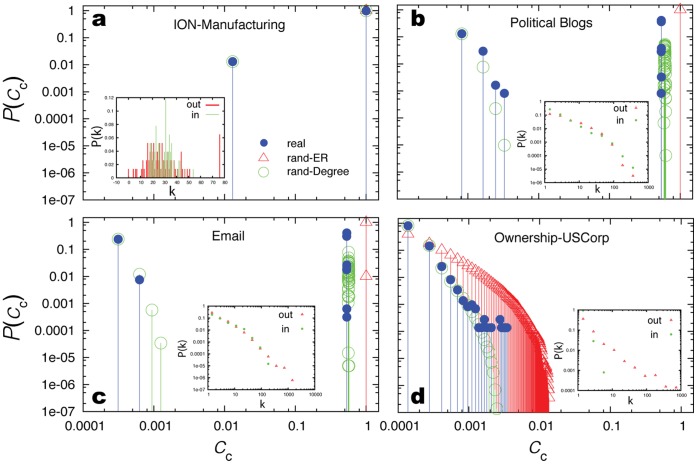
Distribution of normalized control centrality of several real-world networks (blue) and their randomized counterparts: rand-ER (red), rand-Degree (green), plotted in log-log scale. (a) Intra-organizational network of a manufacturing company [49]. (b) Hyperlinks between weblogs on US politics [50]. (c) Email network in a university [51]. (d) Ownership network of US corporations [52]. In- and out-degree distributions for each network are shown in the insets. See Table 1 for other network characteristics.

We first consider the distribution of control centrality. Shown in Fig. 2 is the distribution of the normalized control centrality (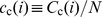) for several real networks. We find that for the intra-organization network, 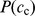 has a sharp peak at 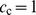, suggesting that a high fraction of nodes can individually exert full control over the whole system (Fig. 2a). In contrast, for company-ownership network, 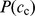 follows an approximately exponential distribution or a very short power-law distribution (Fig. 2d), indicating that most nodes display low control centrality. Even the most powerful node, with 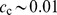, can control only one percent of the total dimension of the system’s full state space. For other networks 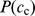 displays a mixed behavior, indicating the coexistence of a few powerful nodes with a large number of nodes that have little control over the system’s dynamics (Fig. 2b,c). Note that under full randomization, turning a network into a directed Erdös-Rényi (ER) random network [35], [36] with number of nodes (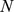) and number of edges (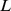) unchanged, the 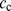 distribution changes dramatically. In contrast, under degree-preserving randomization [37], [38], which keeps the in-degree (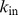) and out-degree (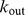) of each node unchanged, the 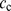 distribution does not change significantly. This result suggests that 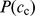 is mainly determined by the underlying network’s degree distribution 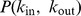. (Note that similar results were also observed for the minimum number of driver nodes [12] and the distribution of control range [28].) This result is very useful in the following sense: 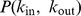 is easy to calculate for any complex network, while the calculation of 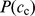 requires much more computational efforts (both CPU time and memory space). Studying 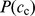 for model networks of prescribed 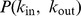 will give us qualitative understanding of how 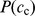 changes as we vary network parameters, e.g. mean degree 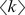. See Fig. S7 for more details.

**Table 1:** Real networks analyzed in the paper.

name	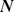	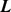	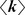	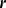	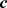
ION-Manufacturing [49]	77	2,228	57.9	−0.017	0.244
Political blogs [50]	1,224	19,025	31.1	−0.196	0.174
Email network [51]	3,188	39,256	24.6	−0.240	0.128
Ownership-USCorp [52]	7,253	6,726	1.9	−0.181	0.004

For each network, we show its name and reference; number of nodes (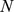) and edges (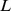); mean degree (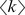); degree correlation (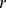) [4]; and clustering coefficient (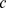) [53], respectively.

### Control Centrality and Topological Features

To understand which topological features determine the control centrality itself, we compared the control centrality for each node in the real networks and their randomized counterparts (denoted as rand-ER and rand-Degree). The lack of correlations indicates that both randomization procedures eliminate the topological feature that determines the control centrality of a given node (see Fig. S2). Since accessibility plays an important role in maintaining structural controllability [29], we conjecture that the control centrality of node 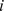 is correlated with the number of nodes 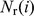 that can be reached from it. To test this conjecture, we calculated 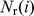 and 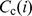 for the real networks shown in Fig. 2, observing only a weak correlation between the two quantities (see Fig. S3). This lack of correlation between 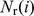 and 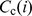 is obvious in a directed star, in which a central hub (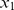) points to 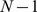 leaf nodes (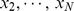) (Fig. 1c). As the central hub can reach all nodes, 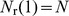, suggesting that it should have high control centrality. Yet, one can easily check that the central hub has control centrality 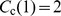 for any 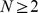 and there are 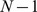 structurally controllable subsystems, i.e. 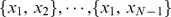. In other words, by controlling the central hub we can fully control each leaf node individually, but we cannot control them collectively.

Note that in a directed star each node can be labeled with a unique *layer index*: the leaf nodes are in the first layer (bottom layer) and the central hub is in the second layer (top layer). In this case the control centrality of the central hub equals its layer index (see Fig. 1c). This is not by coincidence: we can prove that for a directed network containing no cycles, often called a directed acyclic graph (DAG), the control centrality of any node equals its *layer index.*

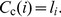
(4)


Indeed, lacking cycles, a DAG has a unique *hierarchical structure*, which means that each node can be labeled with a unique layer index (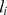), calculated using a recursive labeling algorithm [39]: (1) Nodes that have no outgoing links (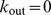) are labeled with layer index 1 (bottom layer). (2) Remove all nodes in layer 1. For the remaining graph identify again all nodes with 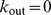 and label them with layer index 2. (3) Repeat step (2) until all nodes are labeled. As the DAG lacks cycles, each subgraph in the set 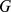 of the directed graph 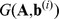 consists of a stem only, which starts from the input node pointing to the state node 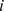 and ends at a state node in the bottom layer, e.g. 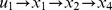 in Fig. 1d. The number of edges in this stem is equal to the layer index of node 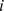, so 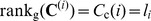. Therefore in DAG the higher a node is in the hierarchy, the higher is its ability to control the system. Though this result agrees with our intuition to some extent, it is surprising at the first glance because it indicates that in a DAG the control centrality of node 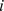 is only determined by its topological position in the hierarchical structure, rather than any other importance measures, e.g. degree or betweenness centrality. This result also partially explains why driver nodes tend to avoid hubs [12]. (Note that similar phenomena about have been observed in other problems, e.g. networked transportation [40], synchronization [41] and epidemic spreading [42]).

Despite the simplicity of Eq. (4), we cannot apply it directly to real networks, because most of them are not DAGs. Yet, we note that any directed network has a underlying DAG structure based on the strongly connected component (SCC) decomposition (see Fig. S4). A subgraph of a directed network is *strongly connected* if there is a directed path from each node in the subgraph to every other node. The SCCs of a directed network 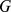 are its maximal strongly connected subgraphs. If we contract each SCC to a single supernode, the resulting graph , called the *condensation* of 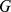, is a DAG [43]. Since a DAG has a unique hierarchical structure, a directed network can then be assigned an underlying hierarchical structure. The layer index of node 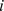 can be defined to be the layer index of the corresponding supernode (i.e. the SCC that node 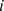 belongs to) in . With this definition of 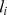, it is easy to show that 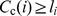 for general directed networks (see Fig. S6 for more details). Furthermore, for an edge 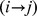 in a general directed network, if node 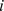 is topologically “higher” than node 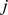 (i.e. 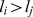), then 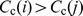. Since 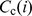 has to be calculated via linear programming which is computationally more challenging than the calculation of 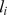, the above results suggest an efficient way to calculate the lower bound of 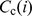 and to compare the control centralities of two neighboring nodes. Note that if 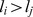 and there is no directed edge 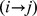 in the network, then in general one cannot conclude that 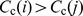 (see Fig. S5 for more details).

### Attack Strategy

Our finding on the relation between control centrality and hierarchical structure inspires us to design an efficient attack strategy against malicious networks, aiming to affect their controllability. The most efficient way to damage the controllability of a network is to remove all input nodes 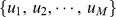, rendering the system completely uncontrollable. But this requires a detailed knowledge of the control configuration, i.e. the wiring diagram of 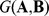, which we often lack. If the network structure (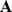) is known, one can attempt a *targeted attack*, i.e. rank the nodes according to some centrality measure, like degree or control centrality, and remove the nodes with highest centralities [44], [45]. Though we still lack systematic studies on the effect of a targeted attack on a network’s controllability, one naively expects that this should be the most efficient strategy. But we often lack the knowledge of the network structure, which makes this approach unfeasible anyway. In this case a simple strategy would be *random attack*, i.e. remove a randomly chosen 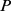 fraction of nodes, which naturally serves as a benchmark for any other strategy. Here we propose instead a *random upstream attack* strategy: randomly choose a 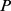 fraction of nodes, and for each node remove one of its incoming or upstream neighbors if it has one, otherwise remove the node itself. A *random downstream attack* can be defined similarly, removing the node to which the chosen node points to. In undirected networks, a similar strategy has been proposed for efficient immunization [45] and the early detection of contagious outbreaks [46], relying on the statistical trend that randomly selected neighbors have more links than the node itself [47], [48]. In directed networks we can prove that randomly selected upstream (or downstream) neighbors have more outgoing (or incoming) links than the node itself. Thus a random upstream (or downstream) attack will remove more hubs and more links than the random attack does. But the real reason why we expect a random upstream attack to be efficient in a directed network is because 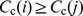 for most edges 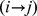, i.e. the control centrality of the starting node is usually no less than the ending node of a directed edge (see Fig. S8). In DAGs, for any edge 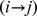, we have strictly 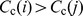. Thus, the upstream neighbor of a node is expected to play a more important or equal role in control than the node itself, a result deeply rooted in the nature of the control problem, rather than the hub status of the upstream nodes.

**Figure 3:**
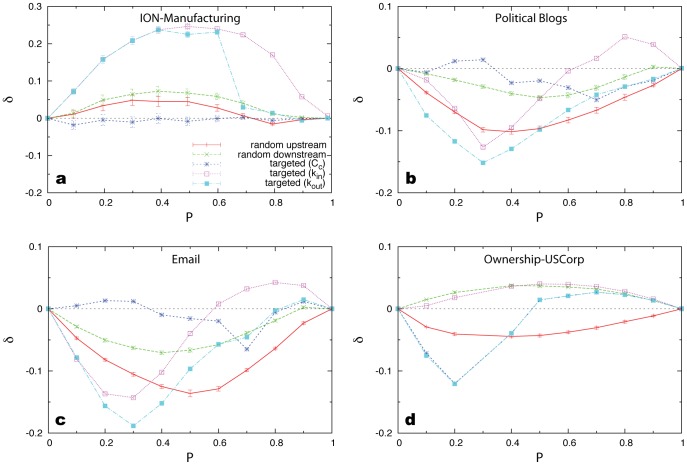
The impact of different attack strategies on network controllability with respective to the random attack. 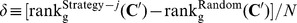
 with 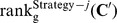 represents the generic dimension of controllable subspace after removing a 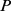 fraction of nodes using strategy-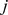. The nodes are removed according to six different strategies. (Strategy-0) Random attack: randomly remove 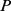 fraction of nodes. (Strategy-1 or 2) Random upstream (or downstream) attack: randomly choose 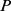 fraction of nodes, randomly remove one of their upstream neighbors (or downstream neighbors). The results are averaged over 10 random choices of 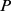 fraction of nodes with error bars defined as s.e.m. Lines are only a guide to the eye. (Strategy-3,4, or 5) Targeted attacks: remove the top 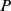 fraction of nodes according to their control centralities (or in-degrees or out-degrees).

To show the efficiency of the random upstream attack we compare its impact on fully controlled networks with several other strategies. We start from a network that is fully controlled (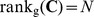) via a minimum set of 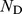 driver nodes. After the attack a 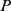 faction of nodes are removed, denoting with 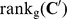 the dimension of the controllable subspace of the damaged network. We calculate 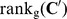 as a function of 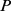, with 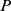 tuned from 0 up to 1. Since the random attack serves as a natural benchmark, we calculate the difference of 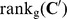 between a given strategy and the random attack, denoted as 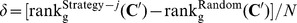. Obviously, the more negative is 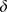, the more efficient is the strategy compared to a fully random attack. We find that for most networks random upstream attack results in 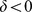 for 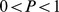, i.e. it causes more damage to the network’s controllability than random attack (see Fig. 3b,c,d). Moreover, random upstream attack typically is more efficient than random downstream attack, even though in both cases we remove more hubs and more links than in the random attack. This is due to the fact that the upstream (or downstream) neighbors are usually more (or less) “powerful” than the node itself.

The efficiency of the random upstream attack is even comparable to targeted attacks (see Fig. 3). Since the former requires only the knowledge of the network’s local structure rather than any knowledge of the nodes’ centrality measures or any other global information (i.e. the structure of the 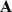 matrix) while the latter rely heavily on them, this finding indicates the advantage of the random upstream attack. The fact that those targeted attacks do not always show significant superiority over the random attacks is intriguing and would be explored in future work. Notice that for the intra-organization network all attack strategies fail in the sense that 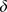 is either positive or very close to zero (Fig. 3a). This is due to the fact this network is so dense (with mean degree 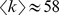) that we have 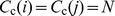 for almost all the edges 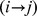. Consequently, both random upstream and downstream attacks are not efficient and the 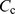-targeted attack shows almost the same impact as the random attack. This result suggests that when the network becomes very dense its controllability becomes extremely robust against all kinds of attacks, consistent with our previous result on the core percolation and the control robustness against link removal [12]. We also tested those attack strategies on model networks (see Fig. S9, S10 and S11). The results are qualitatively consistent with what we observed in real networks.

## Discussion

In sum, we study the control centrality of single node in complex networks and find that it is related to the underlying hierarchical structure of networks. The presented results help us better understand the controllability of complex networks and design an efficient attack strategy against network control. Due to the duality of controllability and observability [18], [19], a similar centrality measure can be defined to quantify the ability of a single node in observing the whole system, i.e. inferring the state of the whole system.

## Supporting Information

Figure S1
**Calculation of control centrality (or the generic dimension of the controllable subspace).** (a) The original controlled system is represented by a digraph 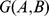. (b) The modified digraph 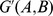 used in solving the linear programming. Dotted and solid lines are assigned with weight 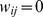 and 1, respectively. The maximum-weight cycle partition is shown in red, which has weight 3, corresponding to the generic dimension of controllable subspace by controlling node 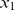 or equivalently the control centrality of node 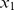.(TIF)

Figure S2 **Control centrality of nodes in several real-world networks and their randomized counterparts: rand-ER (red), rand-Degree (green).** (a) Intra-organizational network of a manufacturing company. (b) Hyperlinks between weblogs on US politics. (c) Email network in a university. (d) Ownership network of US corporations.
(TIF)

Figure S3 **Control centrality vs. the number of reachable nodes.** The real networks are the same as used in Fig. S2.
(TIF)

Figure S4
**Any directed network has a underlying hierarchical structure.** (a) A directed network of 50 nodes. There are seven SCCs highlighted in different colors. The nodes are colored according to their control centrality. The edge 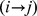 is colored in green, red, or blue if 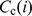 is larger than, smaller than, or equal to 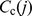, respectively. For all edges with 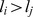, we have 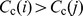. But this is not true for general node pairs 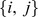. (b) The condensation of the network in (a) is a DAG with three layers. Each node in the DAG represents a SCC in the original network.(TIF)

Figure S5 **Even if a lower node is accessible from a higher node, it is still possible that the control centrality of the higher node is smaller than or equal to the lower one.**
(TIF)

Figure S6
**Control centrality as a function of layer index in several real-world networks.** The real networks are the same as used in Fig. S2. Symbol (‘
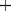
’) represents the average value of 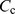 with error bar defined as the 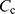 range, i.e. 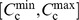, for all the nodes in the same layer of the largest connected component of the network. Dotted lines represents 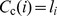.(TIF)

Figure S7
**Variation of the hierarchical structure and its impact on the distribution of control centrality.** (a) Number of layers (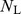). (b) Size of the giant SCC. Both ER and SF networks are generated from the Chung-Lu model with 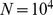 and the results are averaged over 100 realizations with error bars defined as s.e.m. Dotted lines are only a guide to the eye. (c,d,e) Distribution of control centrality for ER networks at different 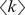 values (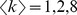).(TIF)

Figure S8
**Fraction of edges** 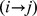 **which satisfy** 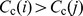. Fractions of edges 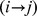 with 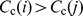, 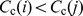, and 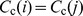, are denoted as 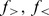, and 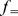, respectively. Both ER and SF networks are generated from the Chung-Lu model with 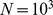 and the results are averaged over 100 realizations with error bars defined as s.e.m. Dotted lines are only a guide to the eye. (a) ER network. (b) SF network with 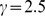. (c) SF network with 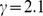.(TIF)

Figure S9
**Impact of different attack strategies on network controllability.** 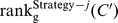 represents the generic dimension of controllable subspace after removing a 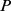 fraction of nodes using strategy-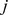. The nodes are removed according to 10 different strategies (see text). Both ER and SF networks are generated from the Chung-Lu model with 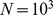 and the results are averaged over 10 random choices of 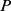 fraction of nodes with error bars defined as s.e.m. Lines are only a guide to the eye.(TIF)

Figure S10
**Impact of different attack strategies on network controllability with respect to random attack.** 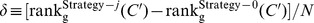 denotes the generic dimension difference of the controllable subspace after removing a 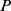 fraction of nodes using strategy-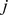 and random attack. The more negative is 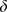, the more efficient is the strategy compared to a random attack. Symbols are the same as used in Fig. S9.(TIF)

Figure S11
**Impact of different attack strategies on network connectivity.** 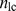 represents the normalized size of the largest connected component of the network after removing a 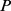 fraction of nodes. The nodes are removed according to 10 different strategies (see text). Both ER and SF networks are generated from the Chung-Lu model with 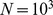 and the results are averaged over 10 random choices of 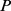 fraction of nodes with error bars defined as s.e.m. Lines are only a guide to the eye.(TIF)
