# Supplementary figures and images for: Control Centrality and Hierarchical Structure in Complex Networks
Source: PLoS One. 2012 Sep 27;7(9):e44459. doi: 10.1371/journal.pone.0044459 (PMC3459977; doi:10.1371/journal.pone.0044459)

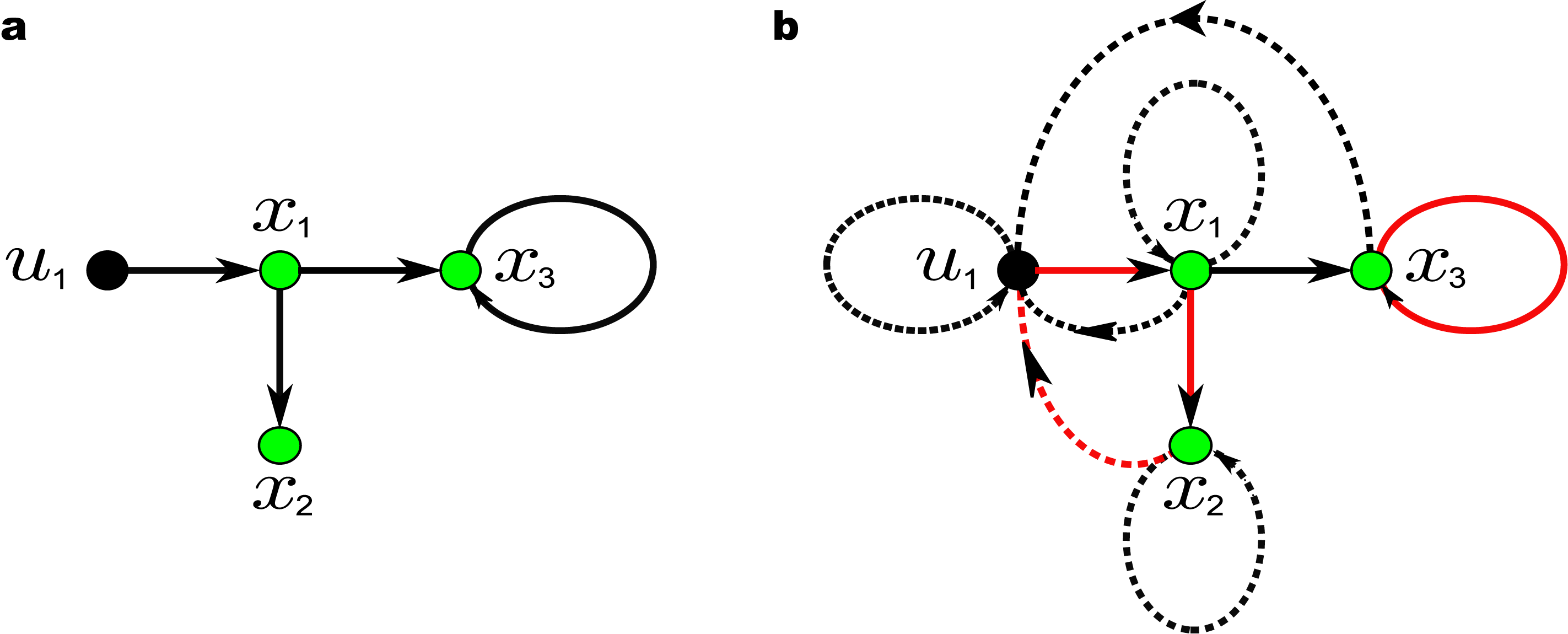

Supplement: Figure S1 — Calculation of control centrality (or the generic dimension of the controllable subspace). (a) The original controlled system is represented by a digraph . (b) The modified digraph used in solving the linear programming. Dotted and solid lines are assigned with weight and 1, respectively. The maximum-weight cycle partition is shown in red, which has weight 3, corresponding to the generic dimension of controllable subspace by controlling node or equivalently the control centrality of node . (TIF) [file pone.0044459.s001.tif]

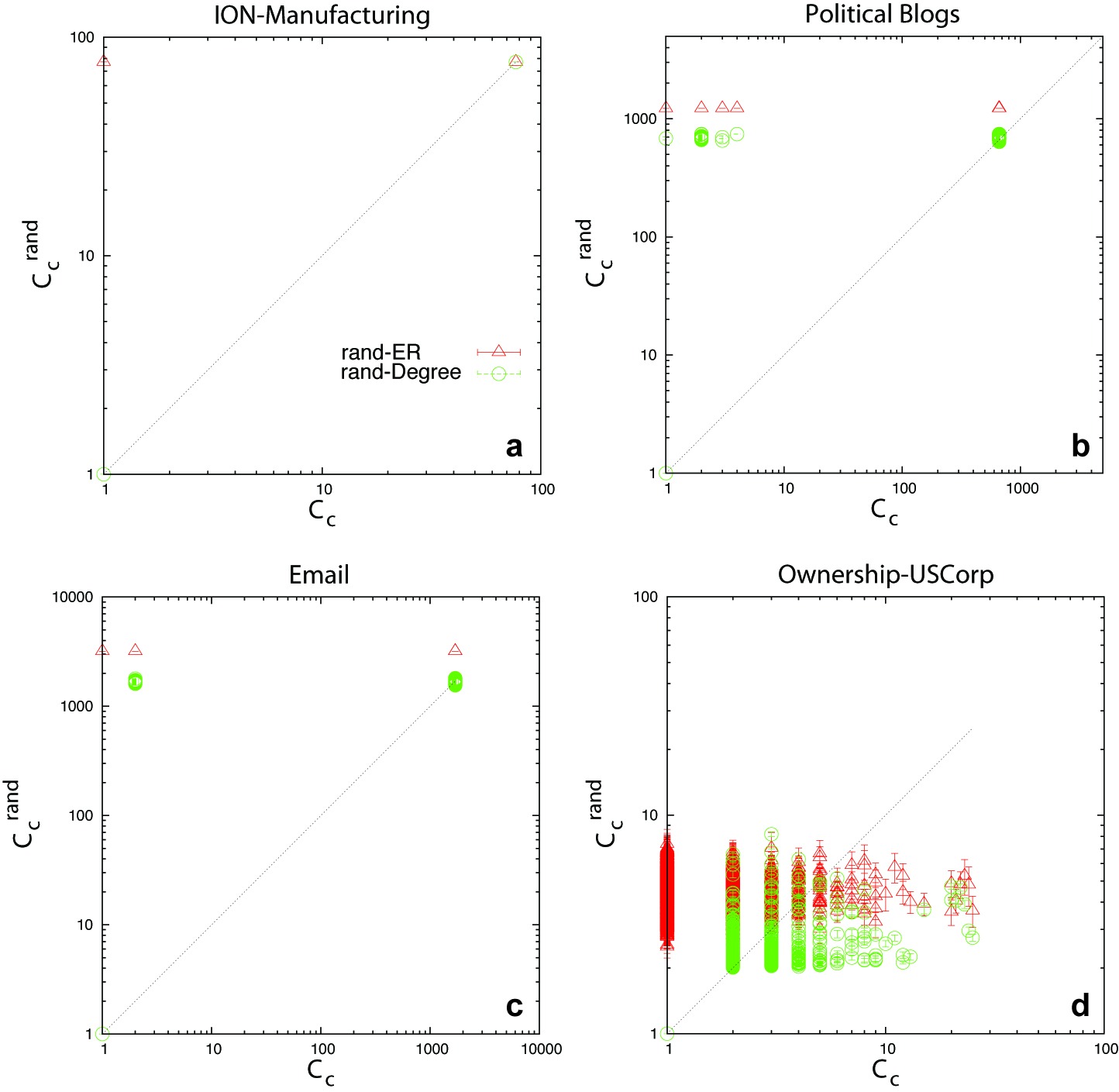

Supplement: Figure S2 — Control centrality of nodes in several real-world networks and their randomized counterparts: rand-ER (red), rand-Degree (green). (a) Intra-organizational network of a manufacturing company. (b) Hyperlinks between weblogs on US politics. (c) Email network in a university. (d) Ownership network of US corporations. (TIF) [file pone.0044459.s002.tif]

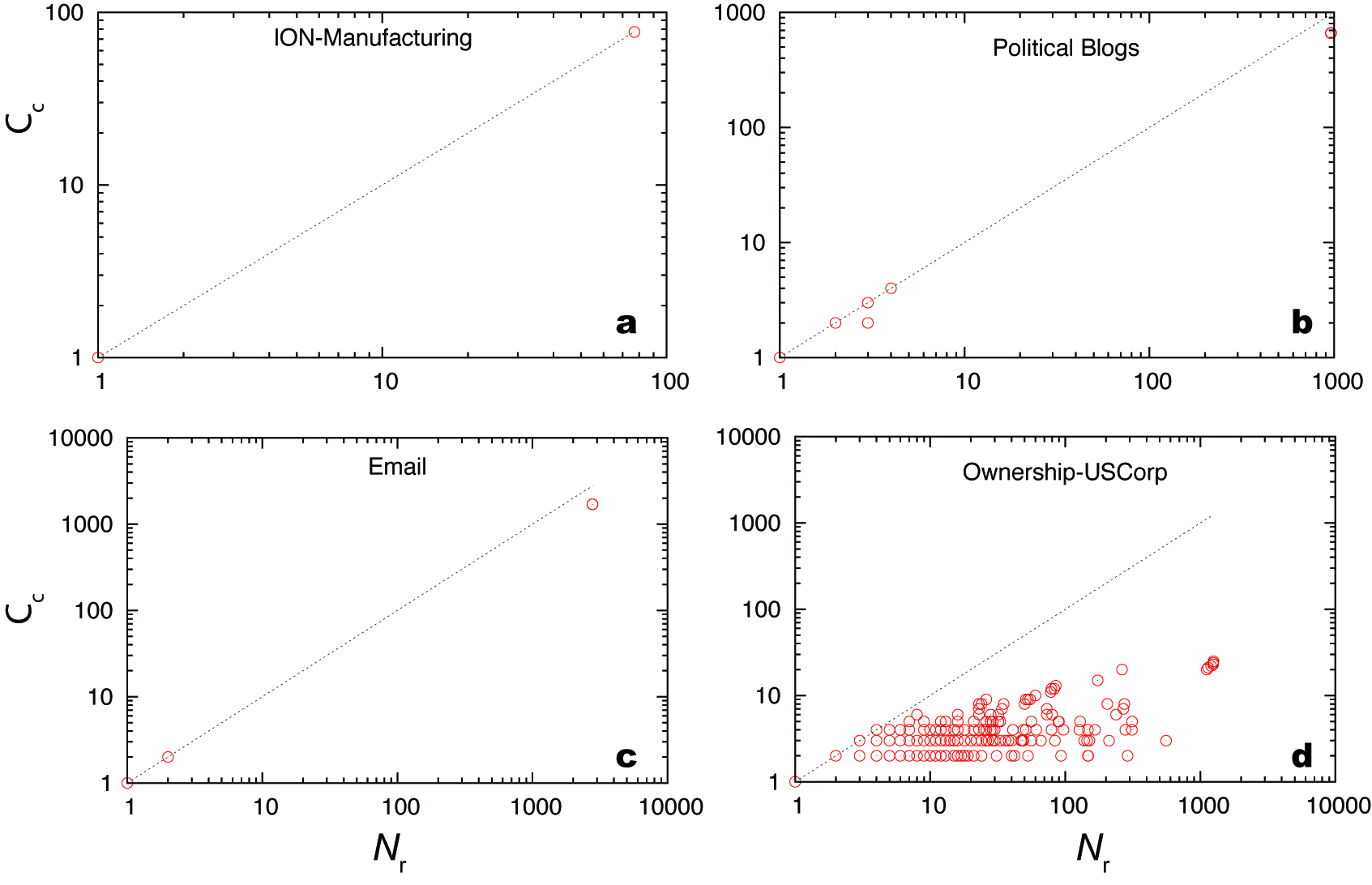

Supplement: Figure S3 — Control centrality vs. the number of reachable nodes. The real networks are the same as used in Fig. S2. (TIF) [file pone.0044459.s003.tif]

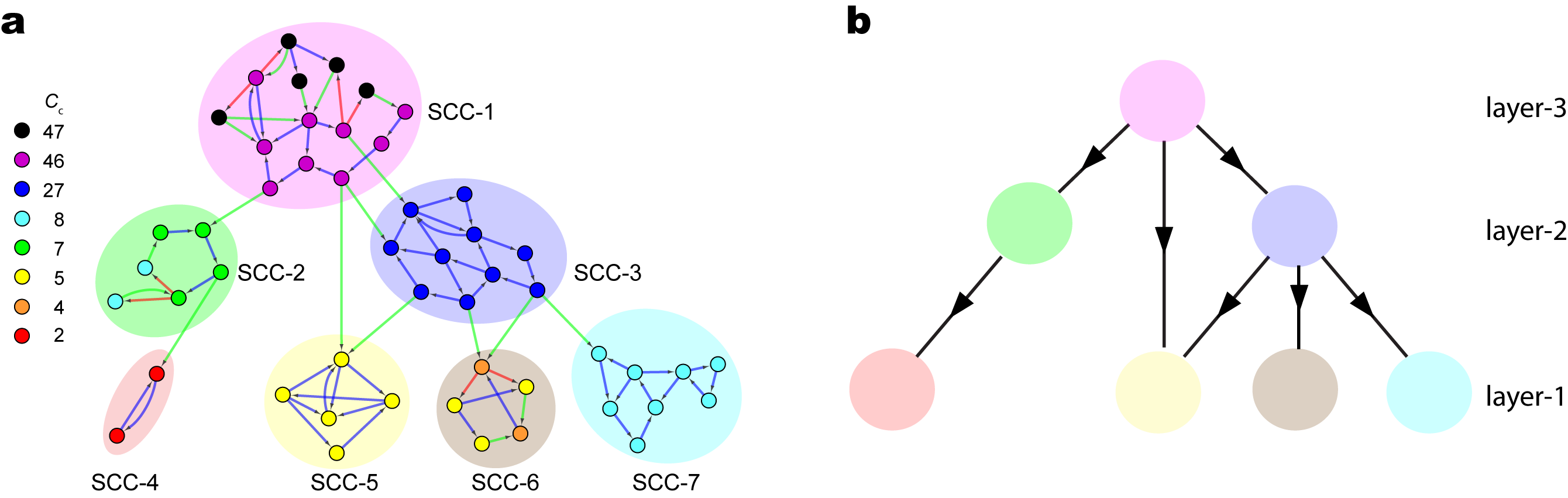

Supplement: Figure S4 — Any directed network has a underlying hierarchical structure. (a) A directed network of 50 nodes. There are seven SCCs highlighted in different colors. The nodes are colored according to their control centrality. The edge is colored in green, red, or blue if is larger than, smaller than, or equal to , respectively. For all edges with , we have . But this is not true for general node pairs . (b) The condensation of the network in (a) is a DAG with three layers. Each node in the DAG represents a SCC in the original network. (TIF) [file pone.0044459.s004.tif]

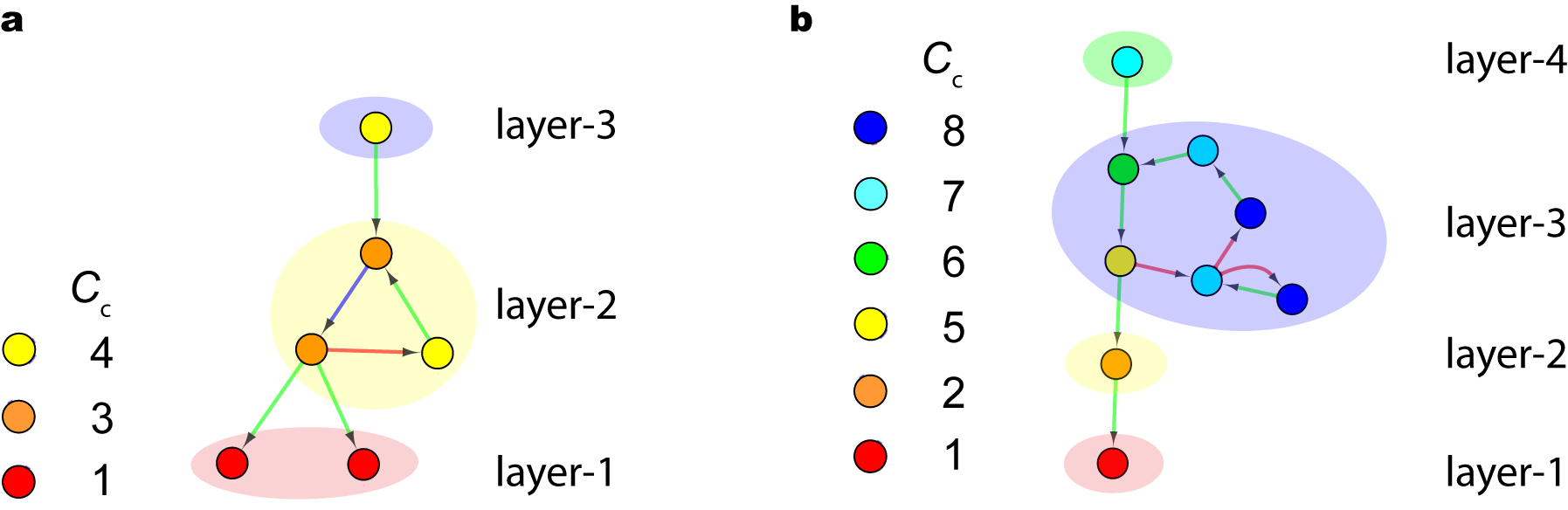

Supplement: Figure S5 — Even if a lower node is accessible from a higher node, it is still possible that the control centrality of the higher node is smaller than or equal to the lower one. (TIF) [file pone.0044459.s005.tif]

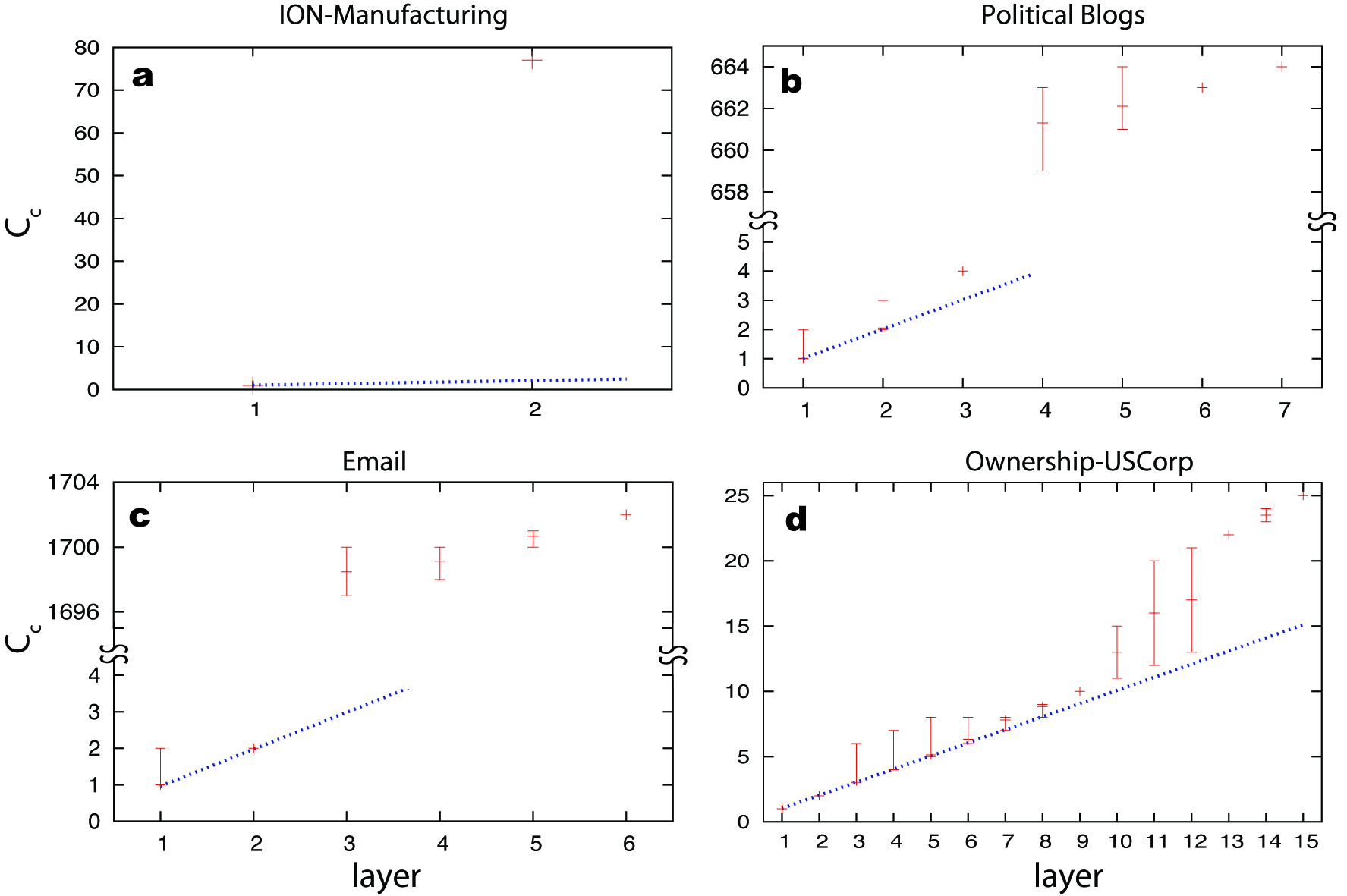

Supplement: Figure S6 — Control centrality as a function of layer index in several real-world networks. The real networks are the same as used in Fig. S2. Symbol (‘’) represents the average value of with error bar defined as the range, i.e. , for all the nodes in the same layer of the largest connected component of the network. Dotted lines represents . (TIF) [file pone.0044459.s006.tif]

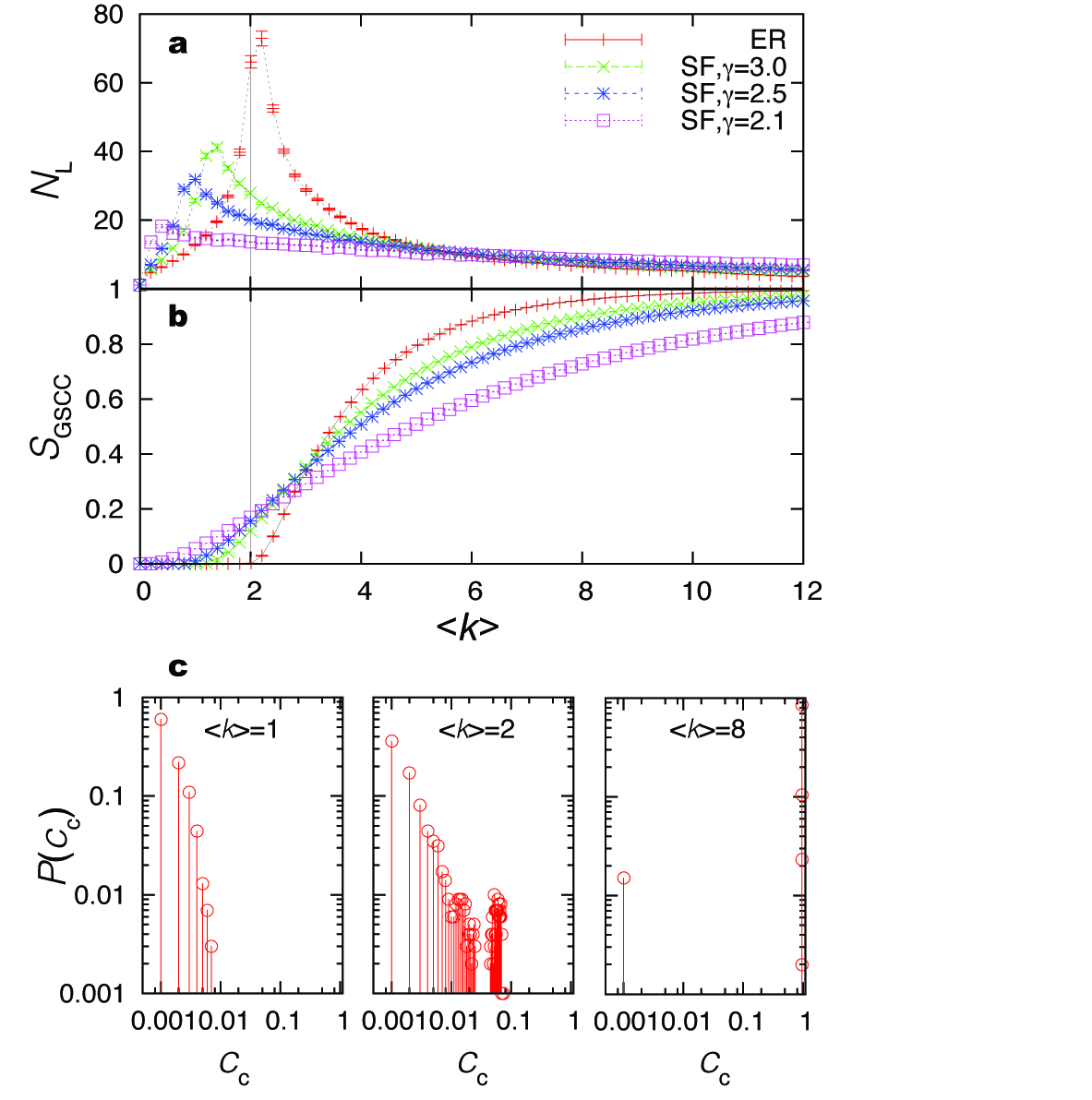

Supplement: Figure S7 — Variation of the hierarchical structure and its impact on the distribution of control centrality. (a) Number of layers (). (b) Size of the giant SCC. Both ER and SF networks are generated from the Chung-Lu model with and the results are averaged over 100 realizations with error bars defined as s.e.m. Dotted lines are only a guide to the eye. (c,d,e) Distribution of control centrality for ER networks at different values (). (TIF) [file pone.0044459.s007.tif]

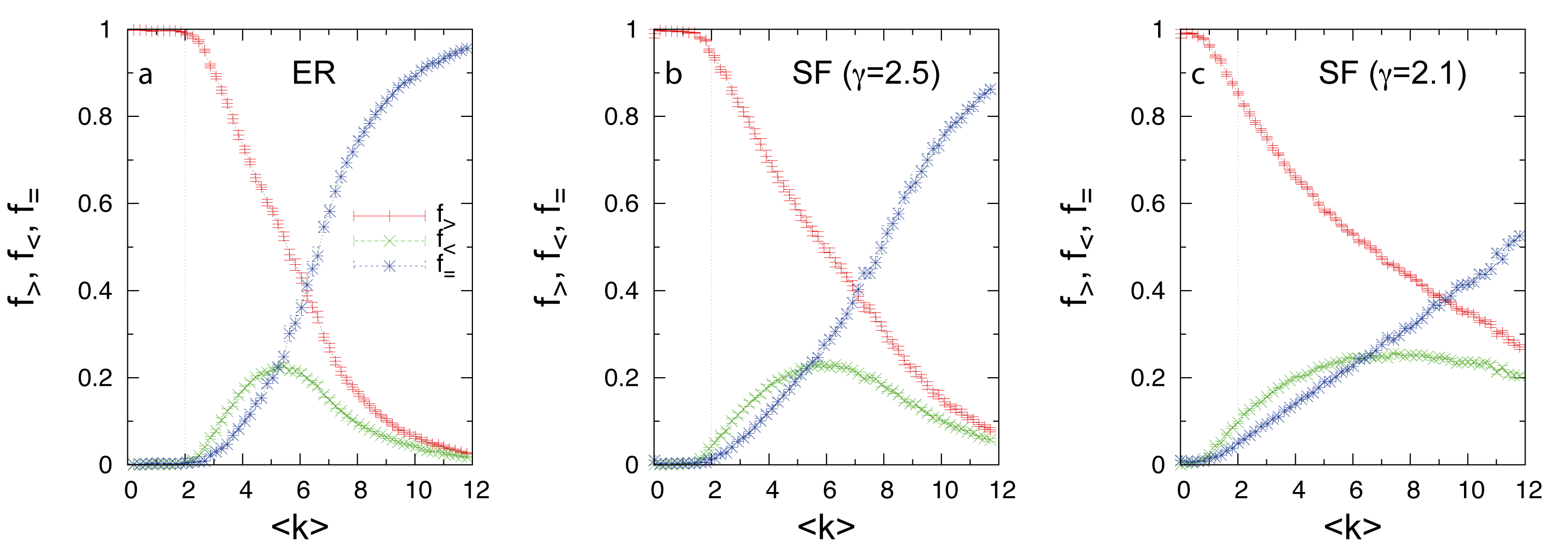

Supplement: Figure S8 — Fraction of edges which satisfy . Fractions of edges with , , and , are denoted as , and , respectively. Both ER and SF networks are generated from the Chung-Lu model with and the results are averaged over 100 realizations with error bars defined as s.e.m. Dotted lines are only a guide to the eye. (a) ER network. (b) SF network with . (c) SF network with . (TIF) [file pone.0044459.s008.tif]

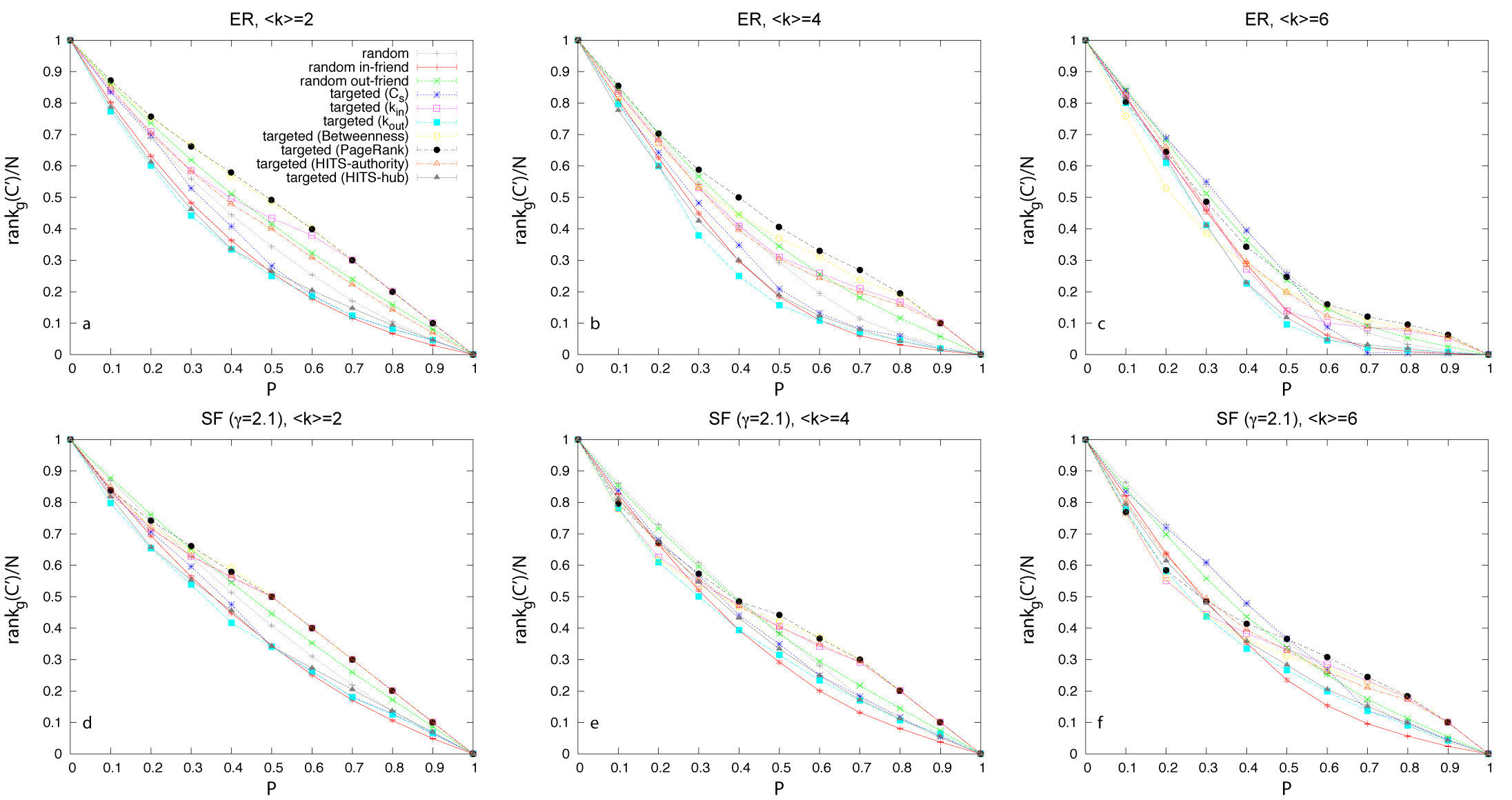

Supplement: Figure S9 — Impact of different attack strategies on network controllability. represents the generic dimension of controllable subspace after removing a fraction of nodes using strategy-. The nodes are removed according to 10 different strategies (see text). Both ER and SF networks are generated from the Chung-Lu model with and the results are averaged over 10 random choices of fraction of nodes with error bars defined as s.e.m. Lines are only a guide to the eye. (TIF) [file pone.0044459.s009.tif]

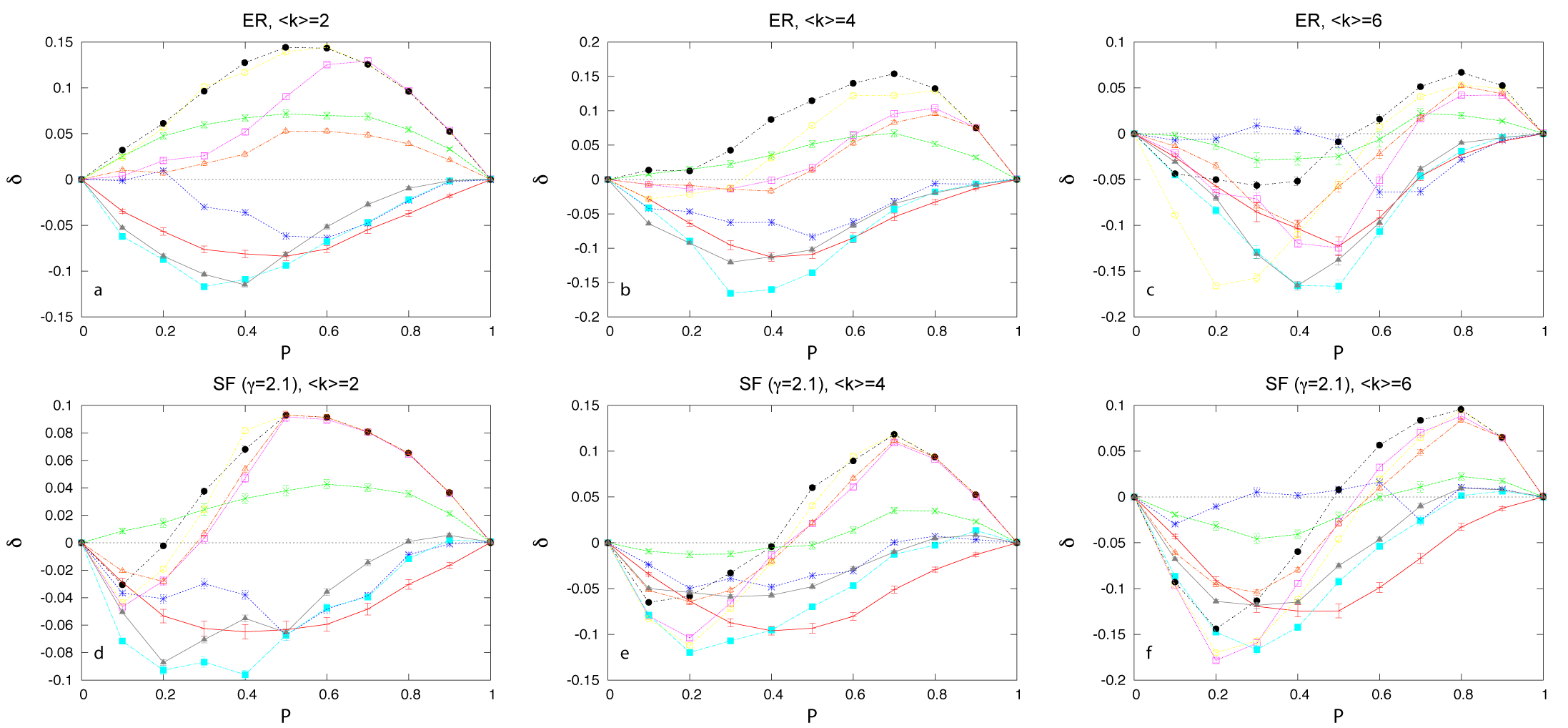

Supplement: Figure S10 — Impact of different attack strategies on network controllability with respect to random attack. denotes the generic dimension difference of the controllable subspace after removing a fraction of nodes using strategy- and random attack. The more negative is , the more efficient is the strategy compared to a random attack. Symbols are the same as used in Fig. S9. (TIF) [file pone.0044459.s010.tif]

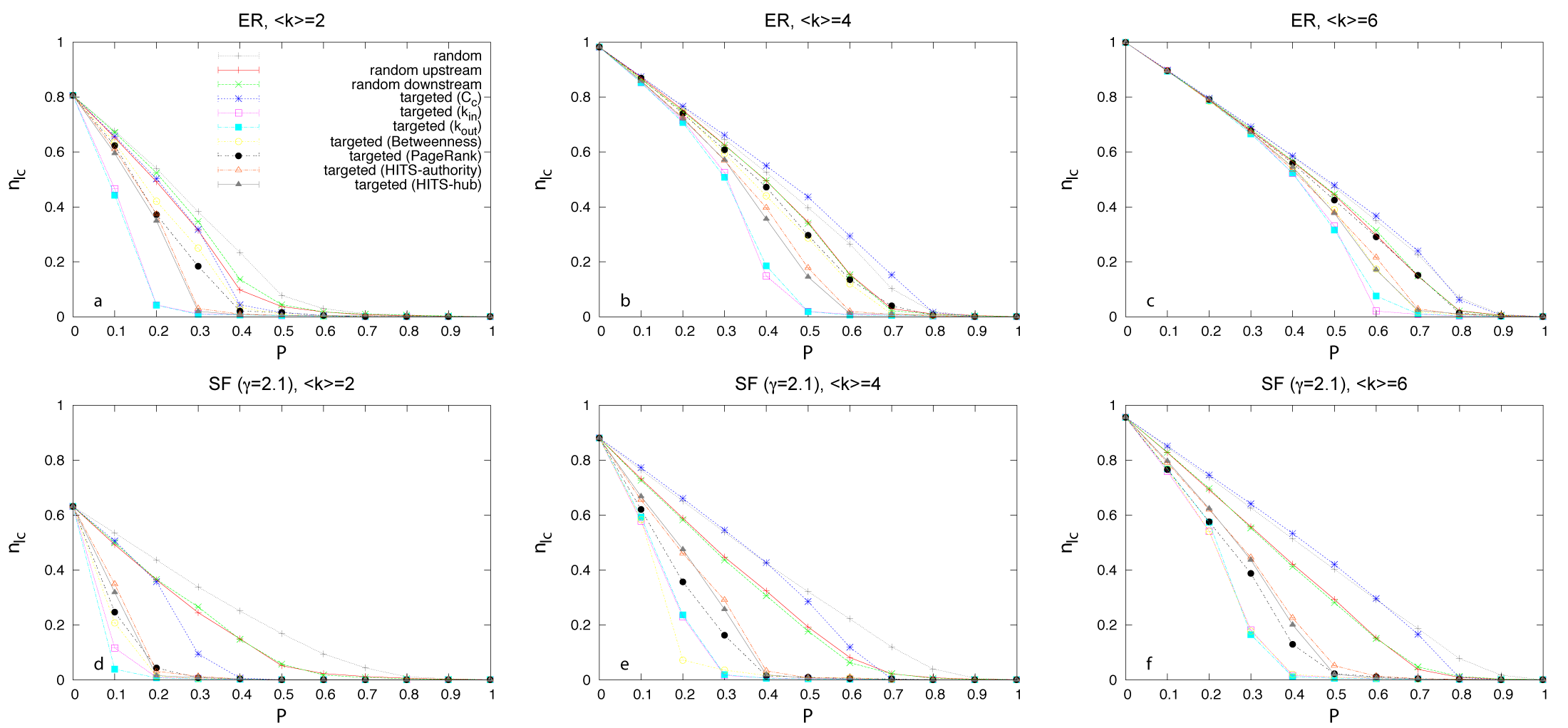

Supplement: Figure S11 — Impact of different attack strategies on network connectivity. represents the normalized size of the largest connected component of the network after removing a fraction of nodes. The nodes are removed according to 10 different strategies (see text). Both ER and SF networks are generated from the Chung-Lu model with and the results are averaged over 10 random choices of fraction of nodes with error bars defined as s.e.m. Lines are only a guide to the eye. (TIF) [file pone.0044459.s011.tif]
